# Fabrication of Poly (Trans-3-(3-Pyridyl)Acrylic Acid)/Multi—Walled Carbon Nanotubes Membrane for Electrochemically Simultaneously Detecting Catechol and Hydroquinone

**DOI:** 10.3390/membranes13070657

**Published:** 2023-07-11

**Authors:** Fabao Luo, Shasha Fan, Maolin Sha, Deshun Cheng, Na Zhang, Chenxiao Jiang, Keying Zhang, Weiguang Fang, Kunyu Ji

**Affiliations:** 1 School of Chemistry and Pharmaceutical Engineering, Hefei Normal University, Hefei 230061, China; fbluo@mail.ustc.edu.cn (F.L.);; 2 Anhui Key Laboratory of Spin Electron and Nanomaterials of Anhui Higher Education Institues, School of Chemistry and Chemical Engineering, Suzhou University, Suzhou 234000, China; 3 School of Physics and Materials Engineering, Hefei Normal University, Hefei 230061, China; 4 Anhui Provincial Engineering Laboratory of Functional Membrane Materials and Technology, School of Chemistry and Materials Science, University of Science and Technology of China, Hefei 230026, China

**Keywords:** Poly(trans-3-(3-Pyridyl)acrylic acid), multi-walled carbon nanotubes, conductive membrane, catechol, hydroquinone

## Abstract

Herein, conductive polymer membrane with excellent performance was successfully fabricated by integrating carboxylated multi-walled carbon nanotubes (MWCNTs) and poly (trans-3-(3-pyridyl) acrylic acid) (PPAA) film. The drop-casting method was employed to coated MWCNTs on the glassy carbon electrode (GCE) surface, and PPAA was then electropolymerized onto the surface of the MWCNTs/GCE in order to form PPAA-MWCNTs membrane. This enables the verification of the excellent performances of proposed membrane by electrochemically determining catechol (CC) and hydroquinone (HQ) as the model. Cyclic voltammetry experiments showed that the proposed membrane exhibited an obvious electrocatalytic effect on CC and HQ, owing to the synergistic effect of PPAA and MWCNTs. Differential pulse voltammetry was adopted for simultaneous detection purposes, and an increased electrochemical responded to CC and HQ. A concentration increase was found in the range of 1.0 × 10^−6^ mol/L~1.0 × 10^−4^ mol/L, and it exhibited a good linear relationship with satisfactory detection limits of 3.17 × 10^−7^ mol/L for CC and 2.03 × 10^−7^ mol/L for HQ (*S/N* = 3). Additionally, this constructed membrane showed good reproducibility and stability. The final electrode was successfully applied to analyze CC and HQ in actual water samples, and it obtained robust recovery for CC with 95.2% and 98.5%, and for HQ with 97.0% and 97.3%. Overall, the constructed membrane can potentially be a good candidate for constructing electrochemical sensors in environmental analysis.

## 1. Introduction

The structural differences of isomers may result in certain differences in their physical and chemical properties. As a result, the accurate identification and detection of isomers by simple and rapid methods has become a challenging and crucial task in the fields of chemistry, biology, pharmacology, and environmental science. Great progress has been made in identifying and detecting isomers, including phenylenediamine isomers, cresol isomers, and eight vitamin E isomers. During phenylenediamine isomers, catechol (CC) and hydroquinone (HQ) are two noteworthy chemical raw materials, which are isomers of each other, and they have similar chemical and physical properties. They are widely used in dye, paint, cosmetics, and other synthetic chemicals in the synthetic manufacturing industry. Studies have proved that CC and HQ are highly toxic to organisms. For example, CC can cause human cancer by destroying DNA [1,2]. The high concentrations of HQ can result in headaches, fatigue, tachycardia, and sometimes even kidney organ damage. Additionally, previous studies have found that phenolic compounds can produce extreme toxicity to aquatic organisms at low concentrations [3], and these compounds do not degrade quickly when entering underground aquifers. CC and HQ in water pose great potential harm to the human body and environment [4,5]. Therefore, it becomes particularly important to exploit a new analytical method with good performance in order to simultaneously discriminate and determine CC and HQ water samples.

Up until now, several conventional analytical techniques, including high-performance liquid chromatography, electrophoresis, gas chromatography, spectrophotometry and electrochemical methods [6,7,8,9,10], have been built. However, most of the above methods, such as high-performance liquid chromatography and spectrophotometry etc., often suffer from complicated operation procedures and expensive equipment, whereas electrochemical techniques show unique advantages in numerous applications due to their low investment cost, their flexible experimental conditions, their simultaneous detection facility without separation being required, and the fact that they are easy to automate. However, electrochemical methods still suffer from a challenge given that the redox peaks of CC and HQ happen to overlap at the surface of the bare electrode. To overcome this problem, various kinds of modified electrodes have been exploited for simultaneously detecting CC and HQ with good performance [11,12,13,14,15]. Among these modified electrodes, the nanomaterials-based modified electrode exhibits excellent qualities and numerous potential applications in the simultaneous detection of CC and HQ. Various nanomaterials were employed to fabricate a modified electrode for determining CC and HQ, such as MgO nanoparticle, Fe_3_O_4_ nanoparticles, reduced graphite oxide, covalent organic frameworks (COFs), gold nanoparticles, carbon nanofibers, carbon nanotubes (CNTs), and so on. For example, the Chen group developed an electrochemical sensor using Pt–MnO_2_ -modified carbon paste electrode for detecting CC and HQ, with potential applications in actual sample detection [16]. The Hu group reported a self-assembly of three-dimensional graphitic carbon nitride (g-C_3_N_4_) nanosheet CNTs composites for electrochemical simultaneous determination of CC and HQ [17]. The Chen group fabricated carbon nanocages-reduced graphene oxide (CN-RGO) composites by the one-pot hydrothermal synthesis method, and they employed it in order to electrochemically simultaneously detect CC and HQ [18]. The Lin group adopted a voltammetry method for determining CC and HQ by employing nitrogen-doped multi-walled carbon nanotubes modified with nickel nanoparticles [19].

Carbon nanotubes, a kind of one dimensional nanomaterial, were discovered in 1991 by Iijima [20], and they have rapidly become a focus of research due to their unique properties, including mechanical, electrical, and chemical ones, as well as their unique tubular molecular structures and their potential applications. Recently, many researchers have paid wide attention to use CNTs as an attractive substrate material to construct electrochemical sensors [21,22,23,24,25]. For example, the Shetti et al. prepared an electrochemical sensor using Ru-TiO_2_/MWCNTs to detect flufenamic acid and mefenamic acid in drugs with excellent performances [26]. Yang et al. fabricated an electrochemical sensor, which used COFs/MWCNT material for the simultaneous detection of CC and HQ. However, the hydrophobicity of CNTs readily aggregates in polar liquids, which hinders its further application in real-world environments. In order to avoid this problem, CNTs are often functionalized with carboxyl groups (CNTs-COOH) on the CNTs surface by strong acids. These oxidized CNTs-COOH have good water solubility. Moreover, the presence of carboxyl groups on the surface of the CNTs provides a chance to link with other compounds by chemical reactions. Although CNTs- COOH possess excellent properties when used as modified material to construct electrochemical sensors, CNTs-COOH-based electrodes still often encounter issues of easy detachment that lead to poor stability, which limits their further application. Thus, it is very important to develop a CNTs-COOH-based electrochemical modified electrode with long-term stability.

Highly conductive membrane electrodes with conductive polymers have received great attention due to the development of electrochemical biosensors. Electropolymerization methods were frequently employed in the synthesis of these conductive polymer coated electrodes. These prepared polymer coated electrodes usually possess good stability, uniformity, and low background current. During the fabrication process of polymer modified electrodes, the coated thickness and other physical properties can be tuned by controlling the polymerization conditions. Various conductive polymer film modified electrodes were employed to detect the CC and HQ. For example, Swamy et al. reported a poly (benzoguanamine) modified electrode for analyzing CC and HQ [27]. Thus, introducing the conductive polymer film into the CNTs-COOH-based electrode would provide a reasonable strategy to develop the HQ and CC electrochemical sensors. In order to utilize the synergistic effect, there have been several studies combining hybrid composites of CNTs-COOH and conductive polymers to prepare electrochemical sensors [28,29]. The hybrid composite CNTs-COOH owns a high electrochemical sensing capability and possible reactive activities, and the conducting polymers can firmly attach CNTs-COOH to the electrode surface. Hence, this quasi-one dimensional structure carboxylated multi-walled carbon nanotubes (MWCNTs-COOH) has been fabricated for applications in electrochemical sensor fields. Wang et al. have reported a hybrid composite film modified electrode coated with MWCNTs-poly(diallyldimethylammonium chloride)-graphene for simultaneously determining CC and HQ, and it has been proved that trans-3(3-pyridyl)allylic acid can be polymerized onto the surface of solid electrode in order to form film with excellent stability and excellent electrocatalytic properties [30].

**Scheme 1:**
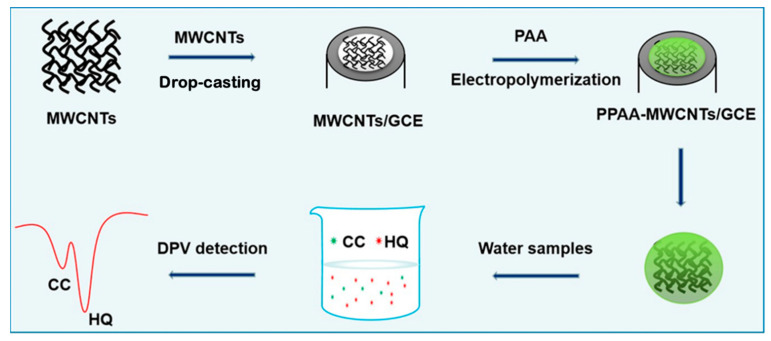
The fabrication process of PPAA-MWCNTs conductive membrane and its application for electrochemical simultaneously detecting HQ and CC.

In this work, a conductive polymer film (PPAA/MWCNTs) was prepared via the electrochemical polymerization method, and it was employed as a modifier to prepare the modified electrode for detecting CC and HQ (Scheme 1). Firstly, MWCNTs was modified onto the bare glassy carbon electrode (GCE) surface by means of the drop-casting method, and trans-3(3-pyridyl)allylic acid(PAA) was then polymerized via the electrochemical method in order to form the PPAA-MWCNTs conductive film on the bare GCE surface. This constructed modified electrode was used to simultaneously analyze the CC and HQ. The electrochemical behaviors of the two isomers on the proposed PPAA-MWCNTs conductive polymer film were investigated by cyclic voltammetry. The electrochemical simultaneously determines the CC and HQ substances in water samples and was carried out by differential pulse voltammetry, and the results indicated that the constructed conductive polymer film could successfully detect the two isomers in the actual water samples with good results. Additionally, the fabricated film exhibited enduring excellent stability and reproducibility, and it may provide a competitive candidate for assaying CC and HQ.

## 2. Results and Discussion

### 2.1. SEM Characterization of PPAA-MWCNTs

**Figure 1:**
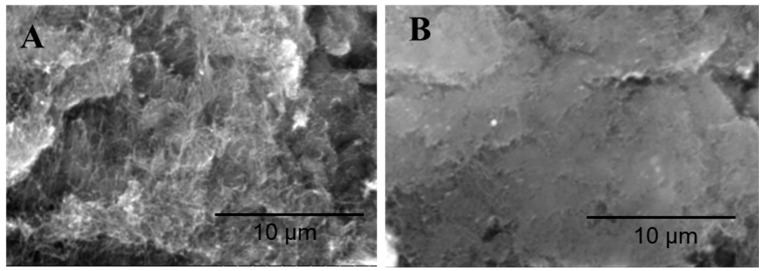
SEM images of (**A**) MWCNTs film and (**B**) PPAA-MWCNTs film. Scale bar: 10 μm.

SEM technique was employed to characterize the morphologies of the MWCNTs film and PPAA-MWCNTs conductive polymer film. In Figure 1A, the long MWCNTs were readily entangled with each other and exhibited local agglomeration, which may be attribute to the interaction of π-π conjugation between the carbon atoms of MWCNTs. As shown in the Figure 1B, after PAA were modified onto the surface of MWCNTs, a thin layer of PPAA polymer film was attached onto the surface of MWCNTs film. The above results indicated that PPAA-MWCNTs conductive polymer film was successfully fabricated, which was similar to the results of a previous report [30].

### 2.2. Electrochemical Behaviors of CC and HQ on the PPAA-MWCNTs/GCE Surface

**Figure 2:**
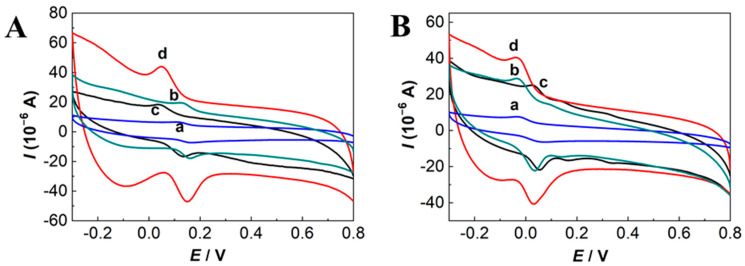
Cyclic voltammetry curves of (**A**) 1.0 × 10^−2^ mol/L CC and (**B**) 1.0 × 10^−2^ mol/L HQ at four different electrodes in 0.1 M PBS (pH = 4.0). (a) GCE; (b) MWCNTs/GCE; (c) PPAA/GCE; (d) PPAA-MWCNTs/GCE.

**Figure 3:**
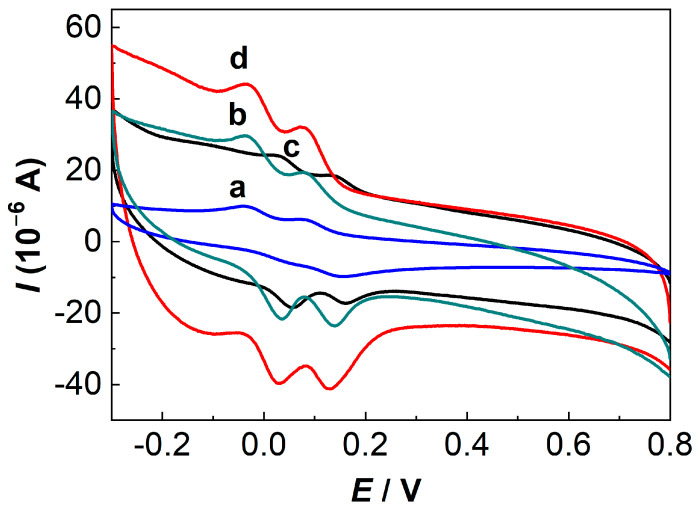
Cyclic voltammetry curves of CC and HQ at four different electrodes. (a) GCE; (b) MWCNTs/GCE; (c) PPAA/GCE; (d) PPAA-MWCNTs/GCE. The PBS was 0.1 M and the pH was 4.0.

The main aim of this work was to construct a method based on the PPAA-MWCNTs conductive polymer film-modified electrode that can achieve simultaneous determination of dihydroxybenzene isomers. From Figure 2 and Figure 3, we can see that our prepared electrodes have extremely high sensitivity and excellent selectivity compared to the conventional bare electrodes for determining the mixture of CC and HQ. In contrast, the two isomers have nearly the same oxidation potential in a voltammetric response at the bare electrode. Figure 2 showed the cyclic voltammetry curves of 1.0 × 10^−2^ mol/L CC and 1.0 × 10^−2^ mol/L HQ in PBS on the different electrodes: bare GCE (a), MWCNTs/GCE(GCE modified with solely MWCNTs) (b), PPAA/GCE(GCE modified with solely PPAA) (c), and PPAA-MWCNTs/GCE (d). It can be seen from Figure 2A that the cyclic voltammetry curves of CC exhibited a pair of weaker redox peaks on the bare electrode, and that its oxidation peak potential (*E_pa_*) and the reduction peak potential (*E_pc_*) was 0.179 V and 0.090 V, respectively, whilst the ∆*E_P_* was 0.089 V. The changes of the oxidation peak and the reduction peak of CC appearing on the MWCNTs film-modified electrode compared with those on the bare electrode were not very obvious. Under the same condition, the cyclic voltammetry curves of CC exhibited an obvious oxidation response and reduction peaks on the PPAA-MWCNTs conductive polymer film modified electrode, and the *E_pa_* and *E_pc_* were 0.146 V and 0.067 V, respectively, whilst the ∆*E_P_* = 0.079 V. Additionally, both the oxidation and reduction peaks currents increased whilst the cathodic and anodic peak potentials negatively shifted, and the above results demonstrated that the PPAA-MWCNTs conductive polymer film modified electrode possessed an excellent electrocatalytic capacity for the oxidation-reduction of CC. As shown in Figure 2B, the ∆*Ep* of HQ on the PPAA-MWCNTs/GCE decreases significantly from 0.109 V to 0.070 V under the same operating conditions, and the transformation of both the oxidation-reduction peaks currents and the cathodic-anodic peak potentials were a similar trend to CC. Additionally, compared with MWCNTs/GCE (b) and PPAA/GCE (c), PPAA-MWCNTs/GCE (d) exhibited a higher electrochemical response to CC and HQ. The above results showed that the PPAA-MWCNTs conductive polymer film exhibited an obvious electrocatalytic effect on the oxidation-reduction of CC and HQ, which may be due to the synergistic effect of PPAA and MWCNTs.

Figure 3 showed that the PPAA-MWCNTs conductive polymer film modified electrode (d) exhibits a well-defined oxidation peak and a reduction peak for HQ and CC, with higher currents than at the bare GCE (a), the MWCNTs film modified electrode (b), and the PPAA/GCE (c). The PPAA in the PPAA-MWCNTs conductive polymer film increased the oxidation peak and the reduction peak currents of the two isomers by approximately twofold compared to the MWCNTs film. The peak currents increase may be due to the larger surface area and the synergistic effect of PPAA and MWCNTs. The above results indicated that the PPAA-MWCNTs conductive polymer film modified electrode could be employed to quantitatively simultaneously determine the CC and HQ substances in the mixture samples.

### 2.3. Effect of Scan Rate and pH

**Figure 4:**
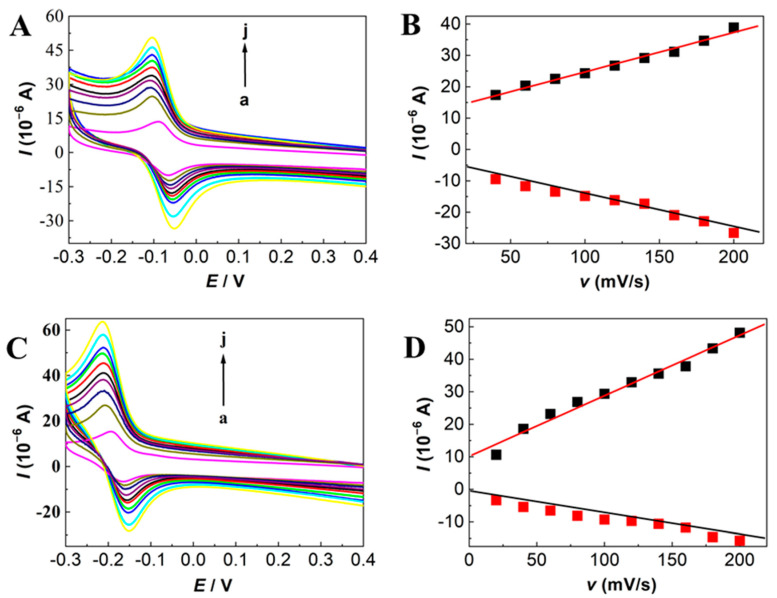
Cyclic voltammetry curves of (**A**) CC and (**C**) HQ at the PPAA-MWCNTs/GCE in 0.1 M PBS (pH = 7.0) with the different scan rate. The corresponding relationship between redox peak currents and scan rate of (**B**) CC and (**D**) HQ. From a to j, the scan rates were 20, 40, 60, 80, 100, 120, 140, 160, 180, and 200 mV/s.

The variation of scan rate at the PPAA-MWCNTs conductive polymer film modified electrode can provide valuable information about electrode kinetics between the solute/solvent interfaces. The detailed analysis of the scan rate effect of CC and HQ was investigated on the PPAA-MWCNTs conductive polymer film modified surface by cyclic voltammetry in 0.1 M PBS (pH = 7.0). From Figure 4A,C, the redox peak current of the CC and HQ increased with the increase in the scan rate during cyclic voltammetry scanning, and a good linear relationship was found between the redox peaks current of CC and the scan rate in the range of 20–200 mV/s. The linear equations were *i_pa_* = 12.12 + 0.13 *v* (R = 0.9933) and *i_pc_* = −5.09–0.10 *v* (R= −0.9855), respectively (shown in Figure 4B). Similarly, as shown in Figure 4D, the redox peak current of HQ in the scan rate range of 20–200 mV/s showed a linear relationship with the scan rate, and the linear equations were *i_pa_* = 10.17 +0.17 *v* (R = 0.9896) and *i_pc_*= −2.50–0.06 *v* (R= −0.9881), respectively. The above results indicated that the reaction process of CC and HQ on the PPAA-MWCNTs conductive polymer film modified electrode surfaces is controlled by adsorption.

**Figure 5:**
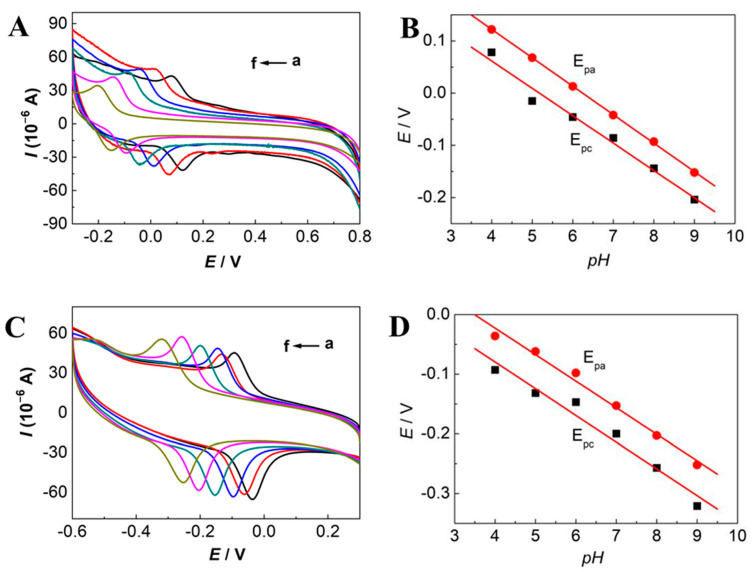
Cyclic voltammetry curves of (**A**) CC and (**C**) HQ at the PPAA-MWCNTs/GCE in 0.1 M PBS with the different pH values, respectively. a = 4.0, b = 5.0, c = 6.0, d = 7.0, e = 8.0, and f = 9.0. The corresponding relationship between redox peak potentials and pH of (**B**) CC and (**D**) HQ.

**Scheme 2:**
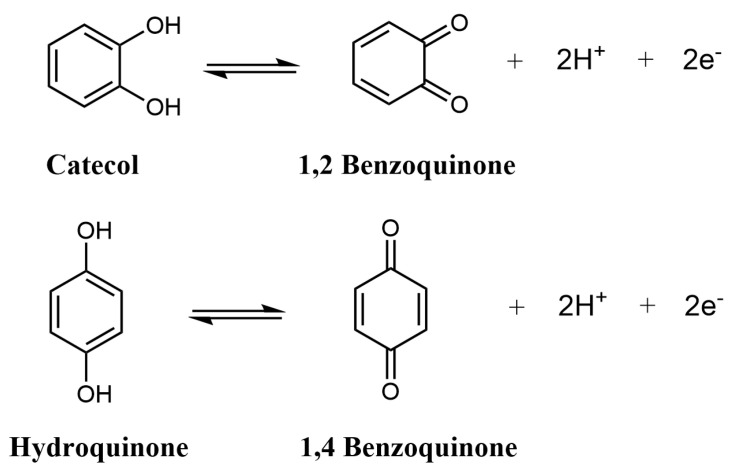
The electrochemical reaction mechanism of HQ and CC at the surface of PPAA- MWCNTs/GCE, respectively.

The supporting electrolyte plays an important role in the electrochemical determination of HQ and CC. The influence of pH on the electrochemical behaviors of the two isomers was investigated by cyclic voltammetry. It can be seen from Figure 5 that the influence of pH (4.0, 5.0, 6.0, 7.0, 8.0, and 9.0) on the peak potentials of the two isomers at the PPAA-MWCNTs conductive polymer film modified electrode was studied via a cyclic voltammetry technique. The tests results revealed that the oxidation peak potential and reduction peak potentials of the CC and HQ in the pH range of 4.0–9.0 gradually shifted negatively with the increase in pH, which suggested that the protons are involved in the oxidation and reduction process of the HQ and CC. The relationship expression in CC was *E_pa_* = 0.3405–0.055 *pH* (R = −0.9998) and *E_pc_* = 0.2717–0.05249 *pH* (R = −0.9900), respectively. Similarly, the relationship expression in HQ was *E_pa_* = 0.1553–0.0445 *pH* (R = −0.9935) and *E_pc_* = 0.09953–0.0448 *pH* (R = −0.9829). According to a previous report [31], the electrochemical reaction mechanism was shown in Scheme 2.

### 2.4. Simultaneous Detection of HQ and CC

**Figure 6:**
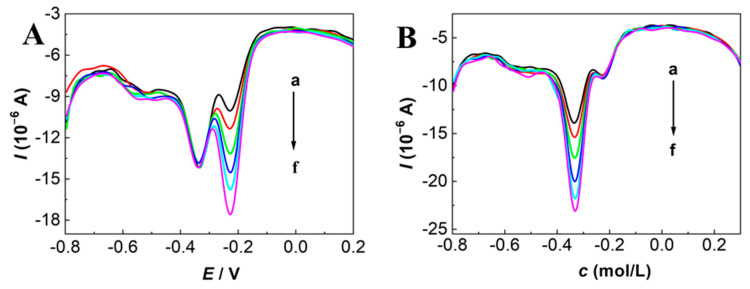
(**A**) Differential pulse voltammetry curves of the different concentrations of CC containing 5.0 × 10^−5^ mol/L HQ at the PPAA-MWCNTs/GCE; (**B**) Differential pulse voltammetry curves of the different concentrations of HQ containing 5.0 × 10^−5^ mol/L CC at the PPAA-MWCNTs/GCE. The PBS was 0.1 M and the pH was 7.0. a = 1.0 × 10^−6^, b = 6.0 × 10^−6^, c = 1.0 × 10^−5^, d = 3.0 × 10^−5^, e = 6.0 × 10^−5^, and f = 1.0 × 10^−4^ mol/L.

The differential pulse voltammetry method in 0.1 mol/L PBS is often used to establish the calibration curve and the detection limit. Hence, the PPAA-MWCNTs conductive polymer film modified electrode for simultaneously sensing CC and HQ in the mixture solution was studied by differential pulse voltammetry. It can be seen from Figure 6A, under containing 5.0 × 10^−5^ mol/L HQ, that the differential pulse voltammetry response increased with the increase in a concentration of CC, and that a good linear response relationship between the oxidation peak current of CC and its concentration in the range of 1.0 × 10^−6^ ~ 1.0 × 10^−4^ mol/L was obtained. The linear regression equation was *i* (10^−6^*A*) = −3.51–0.01c (10^−6^ mol/L) (R = −0.9906). Similarly, it can be seen from the Figure 6B, under containing CC with a concentration of 5.0 × 10^−5^ mol/L, that the differential pulse voltammetry response increased with the increase in the concentration of HQ, and a good linear relationship between the oxidation peak current of HQ and its concentration in the range of 1.0 × 10^−6^~1.0×10^−4^ mol/L was obtained, with the linear regression equation being *i* (10^−6^ *A*) = −6.18–0.08c (10^−6^ mol/L) (R = −0.9937). The above results suggested that the PPAA-MWCNTs conductive polymer film modified electrode may be employed to quantitatively simultaneously determine the substances of the two isomers in mixture samples with satisfactory results.

**Figure 7:**
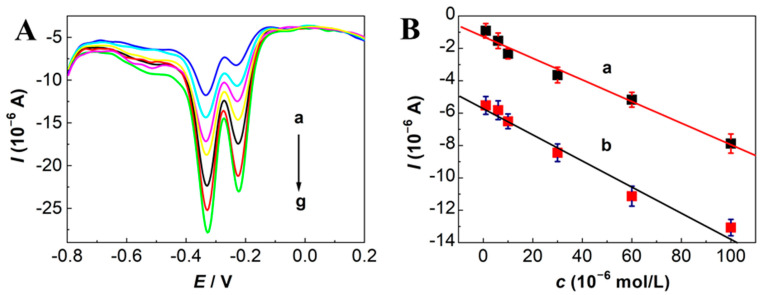
(**A**) Differential pulse voltammetry curves of simultaneous changes in the concentration of CC and HQ at the PPAA-MWCNTs/GCE in 0.1 M PBS (pH = 7.0). (**B**) The concentration of CC (a) and HQ (b) vs. the oxidation peak current. a = 1.0 × 10^−6^, b = 6.0 × 10^−6^, c = 1.0 × 10^−5^, d = 3.0 × 10^−5^, e = 6.0 × 10^−5^, and f = 1.0 × 10^−4^ mol/L.

Figure 7A showed the differential pulse voltammetry curves of the different concentrations of the mixture containing the CC and HQ with a simultaneous concentration increase. The tests showed that the oxidation peak current of CC and HQ increased along with their concentration increasing, and that they both showed good linear relationships when their concentration was in the range of 1.0 × 10^−6^ ~ 1.0 × 10^−4^ mol/L in Figure 7B. The linear regression equations were *i* (10^−6^ *A*) = −1.28–0.07c (10^−6^ mol/L), (R = −0.9924), and *i* (10^−6^ *A*) = −5.70–0.08c (10^−6^ mol/L), (R = −0.9885). The detection limits (*S/N* = 3) were 3.17 × 10^−7^ mol/L for CC and 2.03 × 10^−7^ mol/L for HQ. According to the above results, it can be concluded that the PPAA-MWCNTs conductive polymer film modified electrode can detect CC and HQ selectively or simultaneously. Compared with previous reports [1,32,33,34], as shown in Table 1, the constructed method exhibited a good performance in the linear range and the detection limit. The above results further indicated that the PPAA-MWCNTs conductive polymer film modified electrode provided a potential strategy for quantitative simultaneous determination of the CC and HQ in real samples.

### 2.5. The Stability and Reproducibility

**Figure 8:**
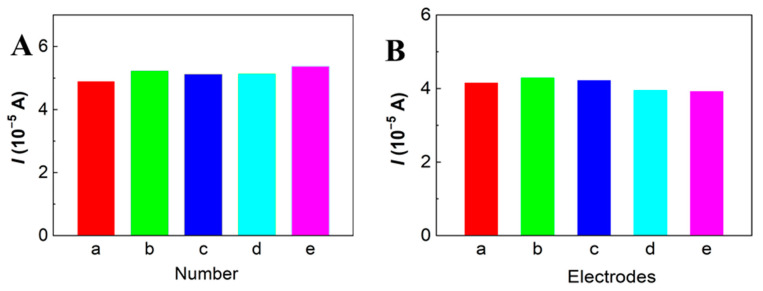
(**A**) The constructed sensor with five times parallel for detecting the same concentration of CC in 0.1 M PBS (pH = 7.0). Detection number. a = 1, b = 2, c = 3, d = 4, and e = 5; (**B**) The constructed sensor with independently fabricating five electrodes for detecting the same concentration of CC in 0.1 M PBS (pH = 7.0). The peak current vs. different electrode.

In order to investigate the practical usage of the proposed electrochemical sensor, several important parameters, including reproducibility and stability, were studied by cyclic voltammetry and the differential pulse voltammetry method. The stability of the proposed electrochemical sensor was evaluated by continuously scanning it for 40 cycles and placing it in 0.1 mol/L PBS (pH = 7.0) for 7 days at 4 °C, and this was then used in order to detect the same concentration of CC. The results showed that the initial peak current was 5.393 × 10^−5^ A after the final electrode was treated, and that the peak current was 5.038 × 10^−5^ A, which allowed for keeping the initial peak currents of 93.42%. The above results indicated that the constructed electrochemical sensor had good stability. The repeatability of this constructed electrochemical sensor was tested by using the PPAA-MWCNTs conductive polymer film modified electrode to determine HQ five times and a relative standard deviation (R.S.D.) of 3.47% was found (as shown in Figure 8A), respectively. Furthermore, the reproducibility of the constructed electrochemical sensor was tested by independently fabricating five electrodes for detecting the same concentration of CC. An R.S.D. of 3.67% was found (as shown in Figure 8B), which implied that the developed method has good reproducibility.

### 2.6. The Real Samples Assay

For the proposed electrochemical sensor, the practical application of the actual sample analysis was the first criterion to judge it good or bad. Herein, we have tested the real applicability of PPAA-MWCNTs/GCE for detecting CC and HQ in different Zhenzhu Lake water samples. The constructed method was adopted in order to analyze the actual sample with the same operation conditions. Before detection, 0.1 M PBS (pH = 7.0) was used to dilute the collected Zhenzhu Lake water. After that, a series of known concentrations of CC and HQ were added to the Zhenzhu Lake water sample via the standard addition method. According to the part of 2.4, the peak current was recorded. The detection amount was calculated via the constructed calibration curve. The added amount of CC and HQ was obtained by the constructed method. After that, the recovery experiments were carried out and then measured. The results were displayed in Table 2. The recoveries were 98.50% and 95.20% for CC and 97.00% and 97.30% for HQ, respectively, which indicated that the proposed sensor could determine CC and HQ in complex samples.

## 3. Materials and Methods

### 3.1. Chemicals and Apparatus

Carboxylated multi-wall carbon nanotubes (MWCNTs) (purity greater than 95%, length 30 mm, diameter 20–30 nm) was purchased from the Zhongke Nano New Material Co., Ltd., Shenzhen, China. Catechol (CC), hydroquinone (HQ), Trans-3-(3-pyridyl) acrylic acid (PAA) was provided by Sinopharm Group Chemical Reagent Co., Ltd. (Beijing, China). In this paper, all analytical pure grade reagents were used without further purification, and all solutions were concocted with double distilled water. Various pH of PBS (Phosphate buffer solutions) were prepared by Na_2_HPO_4_ and NaH_2_PO_4_ solution, and pH value was adjusted through adding H_3_PO_4_ or NaOH solution. All experiments were executed at room temperature conditions.

Electrochemical measurements were operated on a CHI660A electrochemical workstation (Shanghai Chenhua Instruments Co, Shanghai, China). The three electrode system (bare GCE or modified electrode as work electrode, saturated calomel electrode (SCE) as reference electrode, and platinum wire as counter electrode) was adopted. All electrochemical measurements were carried out in a 10 mL electrochemical cell, where O_2_ was removed by bubbling high-purity N_2_ for 15 min. A continuous flow of N_2_ was maintained over the solution to avoid the redissolution of O_2_ during measurements. All potentials given in this paper are referred to SCE. Nitrogen was employed to remove oxygen for 15 min and kept over the solution during the measurements. The scanning electron microscope (SEM) images were obtained via a Hitachi High-Tech SU1510 SEM (Tokyo, Japan).

### 3.2. The Preparation of PPAA-MWCNTs Membrane

Before modification, the bare GCE was polished with aluminum oxide (Al_2_O_3_) powder suspension, followed by ultrasonic washing with 50% ethanol water solution for 1 min, and then allowing for drying naturally at room temperature. A total of 10.0 mg MWCNTs was ultrasonically dispersed in 2 mL water for 30 min to form suspension, 5 μL MWCNTs suspension was then dropped onto the surface of the treated bare GCE, and it was then dried at room temperature to obtain MWCNTs film modified electrode. The MWCNTs film modified electrode was then put into 0.1 mol/L PBS (pH = 7.0), which contained 1.0 × 10^−3^ mol/L PAA. Electropolymerization was carried out through scanning for 20 cycles under the sweep rate of 100 mV/s with a starting potential of −1.2 V and an ending potential of +2.2 V by cyclic voltammetry. After that, the final electrode was washed with double distilled water. The prepared electrode was named PPAA-MWCNTs conductive polymer film modified electrode (PPAA—MWCNTs/GCE).

## 4. Conclusions

In this paper, an excellent PPAA-MWCNTs conductive polymer film modified electrode was prepared by a simple electropolymerization method, and it was employed to simultaneously determine CC and HQ. The tests results showed that the obtained modified electrode was of excellent electrocatalytic ability for the redox of CC and HQ. The constructed detection method was able to achieve simultaneous detection of CC and HQ in mixture, and the detection limits (*S/N* = 3) for CC and HQ were 3.17 × 10^−7^ mol/L and 2.03 × 10^−7^ mol/L, respectively. The constructed membrane showed a good stability and reproducibility. In addition, the PPAA-MWCNTs conductive polymers film-based electrode was easy to operate and had a low detection limit, thereby providing a more efficient method for the determination of CC and HQ in water samples, and, so, it could have a potential application in environmental monitoring fields.

## Figures and Tables

**Table 1:** The comparison of the performances of the different electrodes for the linear range and the detection limit.

Modified Electrode	pH	Linear Range (CC:10^−6^ mol/L)	Detection Limit 10^−6^ mol/L	Linear Range (HQ:10^−6^ mol/L)	Detection Limit 10^−6^ mol/L	Ref.
ML-101(Cr)-rGO-CPE	7.5	10–1400	4.10	4–1000	0.66	[1]
Co_3_O_4_/MWCNTs/GCE	8.0	10–700	8.50	10–800	5.60	[32]
Poly(3,4ethylenedioxythiophene)/CFE	7.0	0.53–86	0.42	0.52–4900	1.60	[33]
Cu-MOFGN/GCE	7.0	1–1000	0.59	1.0–1000	0.33	[34]
PPAA-MWCNTs/GCE	7.0	1.0–100	0.32	1.0–100	0.20	This work

**Table 2:** Determination results of HQ and CC in the water samples.

No.	HQ Added (10^−6^ mol/L)	Found (10^−6^ mol/L)	Recovery (%)	CC Added (10^−6^ mol/L)	Found (10^−6^ mol/L)	Recovery (%)
1	5.00	4.82	97.00	5.00	4.76	95.20
2	10.00	9.73	97.30	10.00	9.85	98.50

## Data Availability

Not applicable.
